# Frailty among rural elderly adults

**DOI:** 10.1186/1471-2318-14-2

**Published:** 2014-01-10

**Authors:** Carmen-Lucia Curcio, Guadalupe-Maria Henao, Fernando Gomez

**Affiliations:** 1 Research Group on Geriatrics and Gerontology, International Association of Gerontology and Geriatrics Collaborative Center, University of Caldas, Manizales, Colombia

**Keywords:** Frailty, Disability, Prevalence, Colombia

## Abstract

**Background:**

This study aimed to estimate the prevalence and associated factors related to frailty, by Fried criteria, in the elderly population in a rural area in the Andes Mountains, and to analyze the relationship of these with comorbidity and disability.

**Methods:**

A cross-sectional study was undertaken involving 1878 participants 60 years of age and older. The frailty syndrome was diagnosed based on the Fried criteria (weakness, low speed, low physical activity, exhaustion, and weight loss). Variables were grouped as theoretical domains and, along with other potential confounders, were placed into five categories: (a) demographic and socioeconomic status, (b) health status, (c) self-reported functional status, (d) physical performance-based measures, and (e) psychosocial factors. Chi-square, ANOVA, and multinomial logistic regression analyses were used to test the prognostic value of frailty for the outcomes of interest.

**Results:**

The prevalence of frailty was 12.2%. Factors associated with frailty were age, gender, health status variables that included self-perceived health and number of chronic conditions, functional covariate variables that included disability in activities in daily living (ADL), disabilities in instrumental ADL, chair stand time, and psychosocial variables that included depressive symptoms and cognitive impairment. Higher comorbidity and disability was found in frail elderly people. Only a subset of frail elderly people (10%) reported no disease or disability.

**Conclusions:**

A relevant number of elderly persons living in rural areas in the Andes Mountains are frail. The prevalence of frailty is similar to that reported in other populations in the Latin American region. Our results support the use of modified Cardiovascular Health Study criteria to measure frailty in communities other than urban settings. Frailty in this study was strongly associated with comorbidities, and frailty and comorbidity predicted disability.

## Background

The scope of frailty in elderly people among minorities is poorly understood. Research on frailty among minority elderly adults and underserved populations is sparse despite evidence of cultural and physiological differences among racial and ethnic groups [1]. Prior analyses need to be extended to larger samples of community-residing adults across several regions, including rural areas.

Although a recent consensus was reached about the domains to evaluate, there has been no agreement on the proposed diagnostic paths and procedures needed to achieve an operational definition [2]. The well-known frailty phenotype by Fried et al. [3] in the Cardiovascular Health Study (CHS), which classifies people into non-frail, pre-frail, and frail categories, has been the most extensively used instrument in different settings. The frailty phenotype postulates that five indicators of physical functioning (unintentional weight loss, exhaustion, slow walking speed, low grip strength, and low physical activity) are related to each other in a cycle of frailty. Several countries have carried out studies based on the frailty criteria developed by Fried et al. [4]. For example, since 2008 several studies have been published in Spain estimating the prevalence of frailty in communities of elderly persons using modified Fried criteria [5-10]. The overall prevalence reported in these studies range from 8.4% to 20.4%. The use of heterogeneous criteria for assessment, characteristics of samples, or measurement analyses is (at least partially) responsible for the diversity of results on this prevalence [11].

A few studies on frailty in elderly adults have been conducted in Latin America. In 2007, based on an extensive revision related to the aging population and frailty, the “Cuban criteria” for frailty were proposed. These criteria included 10 items in several domains, such as the socio-demographic, health, mental status, and psychosocial areas, for use in epidemiological studies [12]. The first epidemiologic study on the prevalence of frailty in the region was the Survey on Health, Well-being and Aging in Latin America and the Caribbean (Salud, Bienestar, & Envejecimiento en America Latina y Caribe, or SABE). This survey involved 7334 adults 60 years of age or older living in five large Latin American and Caribbean (LAC) cities: Bridgetown, Barbados (n = 1446); Sao Paulo, Brazil (n = 1879); Santiago, Chile (n = 1220); Havana, Cuba (n = 1726); and Mexico City, Mexico (n =1063). In this study the prevalence of frailty varied from 30% to 48% in women and from 21% to 35% in men, rates that were much higher than those of their American and European counterparts [13]. In 2009, in a study that followed 4082 community-dwelling elderly adults (mean age of 73 years), a frailty index using 34 variables was developed, which allows stratifying elderly Mexicans into several groups according to the degree of the risk of mortality [14]. The prevalence of frailty has been reported in several studies in the region: Perú, 7.7% [15]; Mexico, 37% [16]; Colombia, 12.1% frail and 53% prefrail [17]; and Brazil, 17.1% frail and 60.1% prefrail [18-20]. In these studies multiple factors were identified with frailty, including advanced age, lower education, presence of comorbidity, poorer self-reported health status, dependence in basic and instrumental activities of daily living (ADL and IADL, respectively), depression, and cognitive impairment [16-21].

The present study aimed to describe the prevalence and related variables of frailty, and to evaluate the relationship between frailty, disability, and comorbidity in a large sample of Colombian, rural, community-dwelling elderly people.

## Methods

### Participants

The study participants included 1692 community-living people 60 years of age and older, living in four villages located in the coffee-growing zone of the Colombian Andes Mountains. The methods of the study have been previously published [22]. The survey was conducted in 2005. Respondents were invited to participate in an ongoing longitudinal database about the risk and protective factors for health in community-living elderly people. They were recruited on a voluntary basis using posters, free sheets, and mass media, including radio and TV publicity.

The study protocol was approved by the human subjects committee of the University of Caldas, Manizales, Colombia. Consent forms were obtained from each respondent. A comprehensive physical assessment was performed on all participants at community centers located in the four villages. Professional health care managers (physicians, nurses, and physical educators), who received intensive instruction on physical performance testing and the survey materials during a two-day training course, carried out the evaluation. It took approximately 30 minutes to complete the assessment. To be included in the study, participants had to be at least 60 years old and able to walk either independently or with an assistive device (4.3% with cane). Participants were excluded based on severe medical conditions (e.g., a physiological unstable disease) or a significant cognitive impairment (e.g., giving no answer to simple identification questions).

### Definition of frailty

All five characteristics from the original phenotype were retained for the present study [3]. However, the measurements used to characterize frailty criteria were slightly different and operationalized as follows:

**Weight loss** was defined as self-reported using Mini-Nutritional Assessment questions [23], unintentional weight loss of 3 kg or more in the previous three months, or as a calculated body mass index (BMI) lower than 21 kg/m^2^, as assessed through anthropometrical measurements [9]. Weight was measured with a SECA precision scale, and height with a stadiometer on a wall without a skirting board.

**Fatigue/exhaustion** was defined by a positive answer to the following question: “In the last two weeks have you suffered from… unwillingness to do things or lack of energy? Or fatigue or tiredness?” [24].

**Slowness** was defined as the lowest quintile in the six-meter walking speed test (range, 0.1 to 1.96 m/s), adjusted for sex and height according to the standards of the Short Physical Performance Battery (less than 0.8 m/sec) [25].

**Weakness** was defined as the lowest quintile of maximum strength on the dominant hand, adjusted for sex and BMI (kg/m^2^) [26]. Strength was measured with a Takey hydraulic dynamometer, the Smedley Hand Dynamometer III.

**Low physical activity** was defined as the lowest quintile in an adapted form of Reuben’s Advanced Activities of Daily Living scale. This scale was created to assess exercise as a physical advanced ADL scale. Responses to three questions were dichotomized to divide people into four categories: frequent non-frail exercisers, frequent long walkers, frequent short walkers, and persons who did not exercise frequently. The last group was defined as the low physical performance group [27].

As recommended earlier [3], participants were classified as frail (3 or more components present), pre-frail (1-2 components), or non-frail (no component).

### Covariates

As detailed elsewhere [17,22], several risk factors for frailty were considered, including those that had been associated with frailty in elderly people in previous studies. Variables were grouped as theoretical domains and placed, along with other potential confounders, into five categories: (a) demographic and socioeconomic status, (b) health status, (c) self-reported functional status, (d) physical performance-based measures, and (d) psychosocial factors.

Demographic characteristics were age, gender, marital status, education, and living arrangements (number of persons living with respondent). Education was measured as years of formal schooling completed (range, 0 to 18). For the analysis, education level was dichotomized (0 to 4 years vs. 5 years or more). Living arrangements (range, 0 to 9) were dichotomized as none (living alone) versus one or more. Socioeconomic status was ascertained by asking the mean individual monthly income. We collapsed individual income categories into a set of two variables reflecting the extreme poverty line (less than $1 per day) or above it as the reference category.

Health status variables included perceived health status, chronic conditions, prevalence of symptoms, medication use, visual and auditory impairment, and cognitive status. Self-perceived health was assessed by asking, “How would you evaluate your present health?” Responses included *very good*, *good*, *fairly*, *bad*, and *very bad*. The last three coding categories were combined for analyses as perceived poor health. The presence of any of seven chronic conditions, namely, arthritis, hypertension, diabetes mellitus, heart disease, stroke, chronic obstructive pulmonary disease, and lower extremities fracture, was ascertained through self-report [28]. Participants were also asked whether they experienced the following symptoms in the last month: memory troubles, breathlessness, and joint or back pain. The number of medications was determined. Polypharmacy was defined as taking four or more medications (including prescribed and not-prescribed medications) [29]. Sensory impairments were assessed by asking for troubles with vision and hearing (yes or no). Cognition was assessed using the Mini-Mental State Examination (MMSE); participants with a score of less than 18 were considered to be cognitively impaired [30].

Information was available regarding self-reported and observed physical function. Self-reported functional status in a physical area was assessed by a Spanish adapted version of the Barthel Index; potential scores were 0 to 100, with a score of 100 considered to be independent [31]. ADL disability was defined as the need for human assistance or the inability to complete the task. Participants with a disability in one or more ADL were determined to have a disability. Self-reported function in IADL was assessed by a Spanish adapted form of the Lawton scale including 13 IADL: preparing meals, walking outside, doing light housework, performing heavy housework, getting to places beyond walking distance, taking medications, turning the radio or TV on and off, turning the lights on and off, opening and closing windows, managing money, managing keys, shopping, and cutting fingernails and toenails. Each item is summed up to produce a scale ranging from 0 to 39, with higher scores considered as independent [32]. IADL disability was defined as having difficulty in or being unable to perform at least one item. Physical performance-based measures included gait speed, rising from a chair, and handgrip strength. We measured chair stand performance by timed rising from a chair two times (range, 0.5 to 9.9 s). We took the mean time for analyses. For the performance-based measures variables, we dichotomized the worst quartile of performance versus the other three quartiles.

Psychosocial function included social participation and depressive symptoms. To assess social participation, we took seven groups of social activities (attending familiar events, trips in the same country, trips abroad, attending religious activities, going to shows or cinema, attending sports events, and participating in groups or volunteering) adapted from the Established for Populations for Epidemiologic Studies of the Elderly interview [33]. Respondents were asked how many times they had done these social activities in the past year. Responses were coded up to 10 or more, with a maximum of 70 (range, 0 to 63). The total score was based on the sum of the items. We collapsed social participation into a set of two variables reflecting the lower quartile versus the other three quartiles and used the lower quartile as the reference category. An abbreviated (score 0 to 15) Spanish-validated Geriatric Depression Scale (GDS-S) was used to assess the presence of depressive symptoms [34], with respondents having a score of 6 or more on the GDS-S considered likely to be depressed. Social support was ascertained if someone could take care of the respondent when the latter became ill (yes or no).

### Statistical analyses

The characteristics of the participants were described by means and standard deviations (SD) or frequencies and percentages according to the type of variable (continuous or categorical, respectively). The chi-square test was used to test qualitative data, while analysis of variance (ANOVA) was used to evaluate continuous data. Statistical differences between groups were determined. A three-step procedure was developed. First, univariate logistic regression analyses were used to describe the unadjusted effect of each of the components of frailty and covariates in the six domains. In the second step, multivariate linear regression models were created to adjust by potential confounder covariates: less than 5 years of education, number of chronic conditions, hypertension, osteoarthritis, heart disease, fractures, stroke, hypercholesterolemia, pain in joints, breathlessness, hearing and visual impairment, polypharmacy, hospitalization in last year, falling last year, injurious falls, fear of falling, disabilities in ADL and IADL, decreased physical activity, gait speed less than 0.82 meters/sec, chair stand less than 1.61 sec, grip strength less than 17 kg, poor perceived health, feeling tired, memory problems, MMSE less than 18, GDS-S more than 6, social participation in less than 10 activities, and poor life satisfaction. Based on previous results, we proceeded with multivariate analysis using multiple multinomial logistic regression, which estimates the prevalence odds ratios (OR) for pre-frail relative to not frail and for frail relative to not frail. To identify the factors associated with frailty, variables were selected based on the strength of the associations, higher prevalence (10% or more), clinical relevance, and low potential for collinearity. We calculated OR and 95% confidence intervals (CI). The p-value for entry into the model was set at p < 0.05. Statistical analyses were performed using SPSS for Windows version 17.0.

## Results

Of the 1,878 participants, 228 (12.2%) were classified as frail, 996 (53%) as pre-frail, and 654 (34.8%) as non-frail. Table 1 shows the respondent characteristics, including demographic, biomedical, and functional variables and psychosocial factors. The mean age of the participants was 70.9 years (SD = 7.4); 52.2% were women and 39% lacked formal schooling. The mean level of education was 3.1 years (SD = 2.8). The mean of comorbidities was 3.21. Disability in performing ADL was reported by 39% of the sample, and disabilities related to mobility ranged from 5.6% for getting in and out of bed or chairs to 9% for climbing stairs. Almost 32% reported at least one fall in the past 12 months. Poor self-perceived health was reported by 18% of the sample, and one third reported increased depressive symptoms. The prevalence of cognitive impairment was 10%.

**Table 1:** Characteristics of the study population

	**Total**	**Men**	**Women**	
**Characteristics**	***n *** **= 1,878**	**n = 897**	**n = 981**	**p value**
	**n (%)**	**n (%)**	**n (%)**	
**Sociodemographics**				
Age in years, mean (SD)	70.9 (7.4)	72.1(7.8)	69.8 (6.8)	<0.001
Age older than 80 years	252 (13.4)	87 (9.7)	165 (16.8)	<0.001
Years of education <5	1268 (70.4)	646 (72)	622 (63.4)	0.059
Poverty	1197 (70.7)	506 (56.4)	524 (53.4)	NS
Living alone	164 (9.4)	91 (10.1)	73 (7.4)	NS
**Comorbidities**				
Number of chronic conditions, mean(SD)	3.21 (1.9)	2.72 (1.7)	3.7 (2)	<0.001
BMI (kg/m^2^),mean(SD)	24.4 (4.5)	23.4 (4.1)	22.5 (4.6)	<0.001
Hypertension	990 (52.7)	411 (45.8)	579 (59)	<0.001
Osteoarthritis	734 (39.1)	309 (34.4)	425 (43.3)	<0.001
Heart disease	373 (19.9)	195 (21.7)	178 (18.1)	NS
Chronic obstructive pulmonary disease	306 (16.3)	96 (10.7)	210 (21.4)	< 0.05
Diabetes mellitus	242 (12.9)	101 (11.3)	141 (14.3)	NS
Stroke	96 (5.1)	45 (5)	51 (5.2)	NS
Lower extremities fractures	219 (11.7)	109(12)	110 (11.2)	NS
Polypharmacy (>4)	284 (15.1)	107 (12)	177 (18)	<0.001
**Symptoms reported**				
Joint pain	618 (32.9)	265 (29.5)	353 (36)	NS
Breathlessness	213 (11.3)	99 (11)	114 (11.6)	NS
Memory problems	628 (33.4)	259 (28.9)	369 (37.6)	NS
**Sensory impairments**				
Hearing	713 (38)	372 (41.5)	330 (34.5)	<0.001
Visual	1293 (68.9)	589 (65.7)	707 (72.1)	<0.001
**Falling**				
At least one fall in past year	599 (32.2)	226 (25.2)	373 (38)	<0.001
Recurrent falls	298 (15.9)	107 (12)	179 (18.2)	NS
Injurious falls in the last year	296 (15.8)	102 (11.4)	194 (19.8)	<0.001
**Functional capacity**				
Disability with ADL	738 (39.3)	310 (34.6)	428 (43.6)	<0.001
Disability with IADL	1188 (63.2)	599 (66.8)	588 (60)	0.002
Gait speed (m/s), mean (SD)	0.95 (0.23)	0.99 (0.25)	0.91 (0.2)	<0.001
Chair stand(s), mean (SD)	1.52 (0.80)	1.52 (0.94)	1.52 (0.65)	NS
Grip strength (kg), mean (SD)	22 (7.36)	26.7 (6.7)	17.8 (4.8)	<0.001
**Psychosocial characteristics**				
Fear of falling	1412 (75.2)	582 (64.9)	830 (84.6)	<0.001
Restriction of activities by fear of falling	796 (51.7)	347 (38.7)	449 (45.8)	NS
Poor perceived health	337 (18)	173 (19.3)	164 (16.7)	NS
Cognitive impairment (MMSE <18, score 0 to 30)	205 (10.9)	103 (11.5)	102 (10.4)	NS
Depression (GDS-S > 6, score 0 to 15))	703 (37.7)	354 (39.5)	349 (35.6)	NS
Social participation (low)	561 (29.9)	315 (35.1)	246 (25.1)	<0.001
Social support not available	413 (22)	222 (24.7)	191 (19.5)	NS

Table 2 provides information on the participants’ characteristics regarding frailty status (non-frail, pre-frail, and frail). Frailty was more frequent in older participants, women, and those with less education. Frail participants had higher comorbidity, basic and instrumental disability, less gait speed, low handgrip strength, and more chair stand time than pre-frail and non-frail participants. Frail participants had more falls than non-frail participants, with lower scores in Barthel ADL and Lawton IADL assessments. Frail elderly adults in the sample were impaired in the MMSE and GDS more frequently than the other two groups.

**Table 2:** Characteristics of the frailty status in the sample

	**Non frail**	**Pre frail**	**Frail**	
**Characteristics**	**n = 654**	**n = 996**	**n = 228**	**p value**
	**(34.8%)**	**(53%)**	**(12.2%)**	
Age in years, mean(*SD*)	69.1 (6.6)	71.4 (7.5)	74 (7.5)	<0.001
Years of education <5	417 (32.9)	671 (52.9)	180 (14.2)	<0.001
Poverty	363 (35.2)	549 (53.3)	118 (14.2)	0.001
Number of chronic conditions, mean(SD)	2.81 (1.8)	3.26 (1.8)	4.1 (2.0)	<0.001
Falling , mean(SD)	1.9 (1.47)	2 (1.69)	2.1 (2.15)	<0.001
Barthel Index, mean(SD)	98 (4.76)	96.5 (6.17)	91.47 (9.98)	<0.001
Lawton Index, mean(SD)	35.7 (4.32)	34.2 (6.15)	29.4 (9)	<0.001
Gait speed (m/s),mean(SD)	1.07 (0.17)	0.93 (0.22)	0.69 (0.22)	<0.001
Chair stand (s), mean(SD)	1.24 (0.28)	1.54 (0.78)	2.24 (1.32)	<0.001
Grip strength (kg/f), mean(SD)	25.8 (6.40)	21.08 (6.9)	15.25 (5.44)	<0.001
Number of medicines, mean(SD)	1.95 (1.7)	2.07 (1.7)	2.16 (1.88)	NS
Hospitalization length of stay last year, mean(SD)	7.42 (13.1)	8.5 (13.3)	7.7 (6.03)	NS
Mini-Mental test Folstein, mean(SD)	25.1 (4.1)	24.1 (4.8)	22.4 (4.7)	<0.001
GDS-S, mean(SD)	3.82 (2.73)	4.85 (2.98)	6.73 (3.12)	<0.001

When CHS original cutoff points were applied, the total participants who met the different frailty criteria numbered 458 (24.4%) for slow walking speed, 435 (23.2%) for weight loss, 429 (22.8%) for weakness, 396 (21.1%) for exhaustion, and 392 (20.9%) for low physical activity. Nine (0.5%) of the cohort participants met five frailty criteria, 44 (2.3%) met four, 175 (9.3%) met three, 368 (19.6%) met two, and 628 (34.0%) met one. Of the 228 participants who were considered frail, CHS criteria were present as follows: low physical activity in 161 (70.6%), exhaustion in 150 (65.8%), weight loss in 97 (42.5%), weakness in 65 (28.5%), and slow walking speed in 53 (23.2%). Statistical differences (p < 0.001) were observed between men and women in weakness, slow walking speed, and low physical activity.

**Figure 1:**
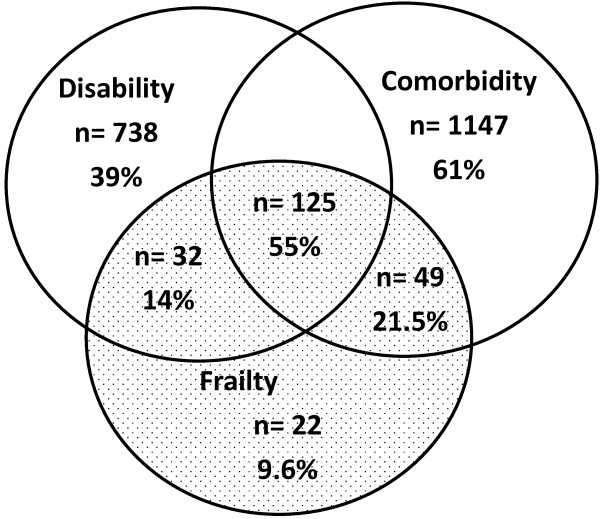
**Interrelationship between frailty (≥3 criteria), disability (≥1 ADL), and comorbidity (≥3 diseases) in the study population.** Venn diagram.

Figure 1 shows the interrelationship between frailty, ADL disability, and comorbidity. One half of frail individuals in the sample reported at least one disability in the ADL scale and three or more comorbidities. Only 9.6% of the frail elderly population reported neither disability nor comorbidities. The covariates by bivariate analysis independently associated with frailty were the following: 80 years of age and older (OR = 2.53, 95% CI 1.54-4.14), female (OR = 2.81, 95% CI 2.06-3.82), less than five years of education (OR = 1.75, 95% CI 1.27-2.41), ADL disability (OR = 4.06, 95% CI 3.01-5.47), high comorbidity (OR = 2.29, 95% CI 1.66-3.16), falls (OR = 1.88, 95% CI 1.42-2.49), disability in ADL (OR = 4.06, 95% CI 2.76-5.98), chair stand time (OR = 6.84,95% CI 5.08-9.34), depressive symptoms (OR = 3.15, 95% CI 2.36-4.21), cognitive impairment (OR = 2.37, 95% CI1.65-3.41), and poor self-perceived health (OR = 3.14 95% CI 2.32-4.28). Table 3 shows the final risk model for frailty as calculated by multivariate multinomial logistic regression and with adjustment for clinical and functional covariates. These results indicate independent associations between age, gender, health status variables (including self-perceived health and number of chronic conditions), functional covariate variables (including disability in ADL), and psychosocial variables (including depressive symptoms and cognitive impairment).

**Table 3:** Adjusted odd ratio (OR) and respective 95% confidence intervals (95%) of the variables associated with pre-frailty and frailty

**Characteristics**	**Pre frailty**		**Frailty**	
	**OR CI (95%)**	**p value**	**OR CI (95%)**	**p value**
Age	1.03 (1.02–1.05)	< 0.01	1.06 (1.03–1.09)	< 0.01
Gender	1.85 (1.46–2.35)	< 0.01	6.16 (2.72–5.94)	< 0.01
Disability (in at least one ADL)	1.28 (1.00–1.63)	0.042	2.55 (1.72–3.79)	< 0.01
Disability in IADL	1.04 (0.82–1.31)	0.18	2.01 (1.26–3.22)	< 0.01
Number of chronic conditions (≥3)	1.03 (0.97–1.10)	0.24	1.18 (1.06–1.31)	< 0.01
Chair stand time	3.22 (2.30–4.52)	< 0.01	5.10 (3.51–7.40)	< 0.01
Cognitive impairment (MMSE <18)	1.33 (0.75–2,36)	0.32	1.90 (1.30–2.77)	0.012
Depression (GDS-S ≥ 6)	1.09 (1.02–1.17)	0.11	1.22 (1.10–1.30)	< 0.01
Poor self-perceived health	1.97 (1.39–2.80)	< 0.01	2.72 (1.67–4.42)	< 0.01

## Discussion

We examined the prevalence of a comprehensive set of risk factors for frailty in elderly people in the rural population of the Andes Mountains in Colombia. To the best of our knowledge, this is the first study to analyze the modified measurements of frailty in rural areas in developing countries, and our results show that frailty is a frequent condition in rural community-dwelling elderly persons. The prevalence of frailty in people 60 years of age and older in rural areas in Colombia was 15.2%, consistent with previous studies that have found the prevalence of frailty to be between 4% and 16.3% [4]. Our results are similar to that of other studies that used comparable methods in Spain [5,10] and Brazil [20] but lower than those in Mexico [16] or in the SABE study [13]. The last study found a higher frequency of frailty (30%-47%), two or three times that of ours. One possible reason for this discrepancy is either the different setting (urban) or the different procedure used in the SABE study to assess the Fried criteria. In general, the prevalence of frailty varies according to the adopted operational definition, the tested population, and the setting where it is explored [11]. The pre-frail prevalence that was found (53%) is consistent with previous studies using the same criteria in similar populations, such as Mexican Americans (47.6%) [35]*,* rural and sub-urban communities in the United States (53.1%) [36], and rural and urban Spanish-speaking populations (42%-45%) [9,10]. However, this prevalence of pre-frailty must be considered a precursor for subsequent frailty, and effective prevention needs to start early.

Few studies have been conducted to measure the prevalence of frailty in elderly persons living in rural areas. A frail/pre-frail prevalence of 14% according to the FRAIL (acronym for Fatigue, Resistance, Aerobic, Illnesses, and Loss of weight) instrument was found among 572 rural community-dwelling persons over 60 years of age from Labastide-Murat in France. When CHS criteria were adopted, the prevalence of frailty increased and showed gender-specific differences [37]. Our results differ concerning this prevalence. One reason for this could be that weight loss, exhaustion, and physical activity are usually self-reported measures, and self-reported measures can be prone to bias related to cultural and socioeconomic differences in thresholds of reporting difficulty [38].

The potential factors associated with frailty in this study were advanced age, female gender, presence of comorbidity, dependence in some basic ADL or IADL, depressive symptoms, cognitive impairment, and negative self-perception of health status. These results are corroborated in the literature [3,5-11,16,20,39]. Of the sociodemographic variables included in the model, age was significantly correlated even when adjusted for the other variables, demonstrating, as in other studies, the influence of the aging process and gender on the emergence of frailty [5-10,16,20]. The higher prevalence of frailty among women has been reported previously [5,7-9,16,20]. It was hypothesized that this excess frailty may be partly due to the marked sex roles still present in this age group of elderly people in rural areas, where most women are housewives with a clear domestic role, restrained social life, and little economic independence, while men are the providers. The association between negative health perception and aging are highlighted in other Latin American frailty studies [16,20]. Thus, we believe that the perception of the elderly regarding adverse experiences during their lifetime may predispose them to frailty [13,16]. The association between frailty and chair stand is in accordance with what has been previously hypothesized, that is, that performance-based measures are significant predictors of frailty (15-21).

Our findings are similar to those of previous studies suggesting that cognitive impairment is strongly associated with frailty [19,21,40]. Recent investigations have reported that being identified as frail is a significant predictor of future cognitive decline [19,21,41]. Some authors have hypothesized that frailty and impaired cognition may share underlying biological causal explanatory factors [19,21,41]. Thus, cognitive impairment has been proposed as part of the frailty phenotype [19,21,41]. A recent cross-sectional survey of 475 adults 70 years of age and older in Mexico, which used modified CHS criteria and added cognitive impairment as another component of frailty, found that cognitive impairment and low physical activity are the main contributing factors of the frailty phenotype to disability [21]. In Brazil, a multicenter and multidisciplinary nationwide effort known as the Network of Studies on the Frailty of Elderly Brazilians (REDE FIBRA), involving 7,983 elderly adults in 17 cities [19], was carried out to collect data on frailty and aging. As a part of this study, 384 community-dwelling elderly adults, 65 years of age and older, in a poor sub-district of the city of São Paulo were assessed to evaluate the association between the CHS frailty criteria and cognition; the study concluded that frailty could be a significant predictor of future cognitive decline [19]. Another pilot study of the same REDE FIBRA involving 391 randomly selected elderly patients aged 65 years, living in Northeast Brazil, reported a prevalence of frailty of 17.1% and pre-frailty of 60.1%. In this study, several factors, including advanced age, presence of comorbidity, dependence in basic ADL and IADL, and negative perception of health, seemed to play an important role in frailty among elderly people [20]. Lastly, we observed that depression (GDS > 6) was present in 36.3% of the frail subjects. This strong association makes sense, since one of the frailty criteria, exhaustion, is part of the diagnostic criteria for depression [8]. The association between depression and frailty is unclear. It is possible that symptoms of depression lead causally to symptoms of frailty and vice versa. Without longitudinal data on both depression and frailty, we cannot determine the predictive relationship between these conditions [42].

The finding of disability in at least one ADL, three or more comorbidities, and three or more frailty criteria in at least one half of participants is higher than that reported in the original CHS study (one of four) or Spanish studies (one of five) [3,8,10,11]. This difference could be due to the high levels of comorbidities and disability that have been reported in the LAC [28]. One reason for these differences is the overall poverty in the LAC. As previously reported, the frailty score was highest for people with lower incomes [18], and disability was more prevalent in the poorest population [28]. In our sample, at least two thirds of the participants were below the poverty line (less than U$1 per day).

Our results are in accordance with recent studies that suggest that frailty is distinct from, but overlapping with, comorbidity and disability [5,9-11,17,20]. Frailty in this study was strongly associated with comorbidities, and frailty and comorbidity predicted disability. Our findings show a greater likelihood of a frail rural elderly person having three or more diseases and being disabled in at least one ADL. Conversely, the observation that a subset of frail elderly people (10%) reported neither disease nor disability is lower than previously reported [6,10]. This finding supports the previous supported hypothesis that when elderly people become frail, there is a final common pathway of severe disease or comorbidity, rather than physiologic changes of aging that are not disease-based [3-18]. This is suggested by our result showing higher rates of poor health status and a greater extent of clinical conditions in the frail group. The relationship between frailty and diseases is poorly understood. In this population, however, individual or comorbid diseases could potentially initiate the frailty process and older people may become disabled earlier than other groups [43]. Moreover, the concept of frailty and mobility disability may largely overlap, as both represent preliminary phases of the disabling process. Therefore, it is possible that some participants presenting mobility disability may not yet experience a complete loss of function in ADL, but may still be frail. Our findings are similar to the results of previous studies suggesting that frailty is strongly associated with disability, but frailty and disability are not the same [3,10,20,35]. In the above-mentioned longitudinal Mexican frailty study, after adjusting for potential confounders, frailty was found to be a predictor of incident mobility disability, and ADL and IADL disability [16].

This study has many strengths, including the number of participants, the comprehensive set of measurements, and the setting of the assessment. To our knowledge, no other study has previously measured the prevalence of frailty in elderly persons living in rural areas in the LAC. Another strength of this study is the comparison with similar studies using CHS criteria in Latin America. Lastly, this study made it possible to establish the relationship between frailty, comorbidity, and disability in a higher prevalence of chronic conditions and disabilities among elderly people in Latin America.

Some limitations of the present study need to be mentioned. Since the present study is cross-sectional, it cannot determine a causal network for frailty. Another limitation is that the cross-sectional nature of this study does not allow strict cause-effect interpretations of the associations between disability, comorbidity, and frailty. Our findings provide information on frailty, prevalence and criteria assessment, and relationships between comorbidity, disability, and frailty in rural populations. Longitudinal studies are needed to specifically explore these relationships.

## Conclusions

In conclusion, our findings provide information on the prevalence of frailty and mobility disability in a rural area in the Andes Mountains. Our results support the use of modified CHS criteria to measure frailty in communities other than urban settings. These results may support the ongoing actions taken by public health authorities aimed at preventing the functional decline of our aging societies. These preliminary data may also help in the design of interventional studies specifically aimed at counteracting the disabling cascade and reversing the frailty syndrome in rural community-dwelling elderly persons.

## Competing interests

The authors declare that they have no competing interests.

## Authors’ contributions

CLS, GMH and FG designed the present study and obtained the funding. CLS, GMH and FG extracted the data, performed the statistical analyses, and wrote the original draft. CLS, GMH and FG revised the draft critically with regard to important intelectual content, and approved the final version of the paper.

## Pre-publication history

The pre-publication history for this paper can be accessed here:

http://www.biomedcentral.com/1471-2318/14/2/prepub

## References

[B1] Yang Y Lee LC Dynamics and heterogeneity in the process of human frailty and aging: evidence from the U.S. older adult population J Gerontol B Soc Sci Psy Sci 2010 14 S246 S255 10.1093/geronb/gbp102 PMC2981448 20007299

[B2] Rodríguez-Mañas L Féart C Mann G Viña J Chatterji S Chodzko-Zajko W Gonzalez-Colaço Harmand M Bergman H Carcaillon L Nicholson C Scuteri A Sinclair A Pelaez M Van der Cammen T Beland F Bickenbach J Delamarche P Ferrucci L Fried LP Gutiérrez-Robledo LM Rockwood K Rodríguez Artalejo F Serviddio G Vega E on behalf of the FOD-CC group Searching for an Operational Definition of Frailty: A Delphi Method Based Consensus Statement. The Frailty Operative Definition-Consensus Conference Project J Gerontol A Biol Sci Med Sci 2013 14 1 62 67 22511289 10.1093/gerona/gls119 PMC3598366

[B3] Fried LP Tangen CM Walston J Newman AB Hirsch C Gottdiener J Seeman T Tracy R Kop WJ Burke G McBurnie MA Frailty in older adults: evidence for a phenotype J Gerontol A BiolSci Med Sci 2001 14 M146 M156 10.1093/gerona/56.3.M146 11253156

[B4] Xue QL The frailty syndrome: definition and natural history Clin Geriatr Med 2011 14 1 15 10.1016/j.cger.2010.08.009 21093718 PMC3028599

[B5] Fernández-Bolaños M Otero A Zunzunegui MV Beland F Alarcón T De Hoyos C Castell MV Sex differences in the prevalence of frailty in a population aged 75 and older in Spain J Am Geriatr Soc 2008 14 2370 2371 10.1111/j.1532-5415.2008.02032.x 19093952

[B6] Alcala MV Puime A Santos MT Barral A Montalvo JI Zunzunegui MV Prevalence of frailty in an elderly Spanish urban population. Relationship with comorbidity and disability Aten Primaria 2010 14 520 527 10.1016/j.aprim.2009.09.024 20116137 PMC7024539

[B7] Abizanda-Soler P Lopez-Torres Hidalgo J Romero Rizos L Lopez Jimenez M Sanchez Jurado PM Atienzar Nuñez P Esquinas JL Garcia I Hernandez P Bardales Y Campos R Martinez M Nieto EO Carion M Ruiz A Aguilar C Manueco P Oliver JL Fragilidad y dependencia en Albacete (FRADEA) razonamiento, diseño y metodologia Rev Esp Geriatr Gerontol 2011 14 81 88 10.1016/j.regg.2010.10.004 21396741

[B8] Abizanda P Sanchez-Jurado PM Romero L Paterna G Martinez-Sanchez E Atienzar-Nuñez P Prevalence of frailty in a Spanish elderly population: the frailty and dependence in Albacete study J Am Geriatr Soc 2011 14 7 1356 1359 10.1111/j.1532-5415.2011.03463.x 21751977

[B9] Jürschik Gimenez P Escobar Bravo MA NuinOrrio C Botigué ST Frailty criteria in the elderly: a pilot study Aten Primaria 2011 14 190 196 10.1016/j.aprim.2010.03.020 20850202 PMC7025926

[B10] Garcia-Garcia F Gutierrez G Alfaro-Acha A Amor MS Lanza MAT Escribano MV Humanes S Larrion JL Gomez-Serranillo M Rodriguez-Artalejo F Rodriguez-Manas L The prevalence of frailty syndrome in an older population from Spain. The Toledo Study for Healthy Aging J Nutr Health Aging 2011 14 10 852 856 10.1007/s12603-011-0075-8 22159772 PMC12878005

[B11] Castell Alcalá MV Melgar Borrego AB Julián Viñals R Canto de Hoyos Alonso M Consideraciones sobre los estudios de prevalencia de fragilidad en el mayor en España Aten Primaria 2011 14 5 295 296 22014854 10.1016/j.aprim.2011.06.008 PMC7025194

[B12] Galban PA Sansó FJ Dìaz-Canel AM Carrasco M Oliva T Envejecimiento poblacional y fragilidad en el adulto mayor Rev Cubana Salud Pública 2007 14 1 1 17

[B13] Alvarado BE Zunzunegui MV Béland F Life course social and health conditions linked to frailty in Latin American older men and women J Gerontol A Biol Sci Med Sci 2008 14 1399 1406 10.1093/gerona/63.12.1399 19126855

[B14] García-González JJ García-Peña C Franco-Marina F Gutierrez-Robledo LM A frailty index to predict the mortality risk in a population of senior Mexican adults BMC Geriatr 2009 14 47 doi: 10.1186/1471-2318-9-47 10.1186/1471-2318-9-47 19887005 PMC2776593

[B15] Varela-Pinedo L Ortiz-Saavedra PJ Chavez-Jimeno H Velocidad de la marcha como indicador de fragilidad en adultos mayores de la comunidad en Lima, Peru Rev Esp Geriatr Gerontol 2010 14 1 22 25 10.1016/j.regg.2009.07.011 20044176

[B16] Aguilar-Navarro S Gutierrez-Robledo LM Garcia-Lara JMA Payette H Amieva H Avila- Funes JA The phenotype of frailty predicts disability and mortality among Mexican community-dwelling elderly J Frailty Aging 2012 14 3 111 117 10.14283/jfa.2012.18 27093198

[B17] Gomez F Curcio CL Henao GM Fragilidad en ancianos colombianos Rev. Medica. Sanitas 2012 14 4 8 16

[B18] Costa TB Neri AL Medidas de atividade física e fragilidade em idosos: dados do FIBRA Campinas, São Paulo, Brasil Cad Saúde Pública 2011 14 8 1537 1550 10.1590/S0102-311X2011000800009 21877002

[B19] Yassuda MS Lopes A Cachioni M Falcao DVS Batistoni SST Guimaraes VV Neri AL Frailty criteria and cognitive performance are related: data from the FIBRA study in Ermelino Matarazzo, Sao Paulo, Brazil J Nutr Health Aging 2012 14 1 55 61 10.1007/s12603-012-0003-6 22238002 PMC12878099

[B20] Sousa ACPA Dias RC Maciel ACC Guerra RO Frailty syndrome and associated factors in community-dwelling elderly in Northeast Brazil Arch Gerontol Geriatr 2012 14 2 e95 e101 10.1016/j.archger.2011.08.010 21930311

[B21] Avila-Funes JA Pina-Escudero SD Aguilar-Navarro S Gutierrez-Robledo LM Ruiz Arregui L Amieva H Cognitive impairment and low physical activity are the components of frailty more strongly associated with disability J Nutr Health Aging 2011 14 8 683 689 10.1007/s12603-011-0111-8 21968865 PMC12878891

[B22] Curcio CL Gomez F Reyes-Ortiz CA Activity restriction related to fear of falling among older people in the Colombian Andes Mountains: are functional or psychosocial risk factors more important? J Aging Health 2009 14 3 460 479 10.1177/0898264308329024 19318606

[B23] Guigoz Y Vellas B Garry PJ Vellas BJ, Guigoz Y, Garry PJ, Albarede JL The Mini Nutritional Assessment (MNA): a practical assessment tool for grading the nutritional state of elderly patients Nutrition in the elderly-the Mini Nutritional Assessment (MNA). Facts research and intervention in geriatrics 1997 Paris: Serdi Publishing 15

[B24] Heikkinnen E Waters WE Brzezinski ZJ The elderly in eleven countries World Health Organization. Regional Office for Europe 1983 Copenhagen: Public Health in Europe 21

[B25] Guralnik JM Simonsick EM Ferrucci L Glynn RJ Berkman LF Blazer DG Scherr PA Wallace RB A short physical performance battery assessing lower extremity function: association with self-reported disability and prediction of mortality and nursing home admission J Geronto 1994 14 2 M85 M94 10.1093/geronj/49.2.M85 8126356

[B26] Curcio CL Gómez JF Fuerza de agarre en los adultos mayores de los centros dia del municipio de Manizales Rev Asoc Colomb Gerontol Geriatr 2005 14 4 849 858

[B27] Reuben DB Laliberte L Hiris J Mor V A hierarchical exercise scale to measure function at the Advanced Activities of Daily Living (AADL) level J Am Geriatr Soc 1990 14 10 855 861 2387949 10.1111/j.1532-5415.1990.tb05699.x

[B28] Menendez J Guevara A Arcia N León-Diaz EM Marin C Alfonso JC Enfermedades crónicas y limitación functional en adultos mayores: estudio comparativo en siete ciudades de América Latina y el Caribe Rev Panam Salud Publica 2005 14 353 361 16053645 10.1590/s1020-49892005000500007

[B29] Rollason V Vogt N Reduction of polypharmacy in the elderly: a systematic review of the role of the pharmacist Drugs Aging 2003 14 11 817 832 10.2165/00002512-200320110-00003 12964888

[B30] Tombaugh TN McIntyre NJ The Mini-Mental State Examination: a comprehensive review J Am Geriatr Soc 1992 14 922 935 1512391 10.1111/j.1532-5415.1992.tb01992.x

[B31] Baztán JJ Pérez del Molino J Alarcón T San Cristóbal E Izquierdo G Manzabeitia I Índice de Barthel: instrumento válido para la valoración funcional de pacientes con enfermedad cerebrovascular Rev Esp Geriatr Gerontol 1993 14 32 40

[B32] Curcio CL Gómez JF Galeano IC Validez y reproducibilidad de las medidas basadas en la ejecución Rev Esp Geriatr Gerontol 2000 14 82 88

[B33] Cornoni-Huntley J Brock DB Ostfeld AM Taylor JO Wallace RB The established populations for the epidemiologic study of the elderly: resource data book 1986 Bethesda, MD: National Institutes of Health NIH No. 86-2443)

[B34] Martí D Miralles R Llorach I García-Palleiro P Esperanza A Guillén J Trastornos depresivos en una unidad de convalecencia: experiencia y validación de una versión española de 15 preguntas de la escala de depresión geriátrica de Yesavage Rev Esp Geriatr Gerontol 2000 14 7 14

[B35] Ottenbacher KJ Graham JE Al Snih S Raji M Samper-Ternent R Ostir GV Markides KS Mexican Americans and frailty: findings from the Hispanic established populations epidemiologic studies of the elderly Am J Public Health 2009 14 4 673 679 10.2105/AJPH.2008.143958 19197079 PMC2661466

[B36] Espinoza SE Hazuda HP Frailty in older Mexican-American and European-American adults: Is there an ethnic disparity? J Am Geriatr Soc 2008 14 1744 1749 10.1111/j.1532-5415.2008.01845.x 18662198

[B37] Cesari M Demougeot L Boccalon H Vellas B Prevalence of frailty and mobility limitation in a rural setting in France J Frailty Aging 2012 14 4 169 173 10.14283/jfa.2012.26 27093317

[B38] Melzer D Lan TY Tom BDM Deeg DJH Guralnik JM Variation in thresholds for reporting mobility disability between national population subgroups and studies J Gerontol A Biol Sci Med Sci 2004 14 12 1295 1303 10.1093/gerona/59.12.1295 15699529

[B39] Santos-Eggimann B Cuenoud P Spagnoli J Junod J Prevalence of frailty in middle aged and older community-dwelling Europeans living in 10 countries J Gerontol A Biol Sci Med Sci 2009 14 6 675 681 19276189 10.1093/gerona/glp012 PMC2800805

[B40] Avila-Funes JA Amieva H Barberger-Gateau P Le Goff M Raoux N Ritchie K Cognitive impairment improves the predictive validity of the phenotype of frailty for adverse health outcomes: the three-city study J Am Geriatr Soc 2009 14 453 461 10.1111/j.1532-5415.2008.02136.x 19245415

[B41] Samper-Ternent R Al Snih S Raji MA Markides KS Ottenbacher KJ Relationship between frailty and cognitive decline in older Mexican Americans J Am Geriatr Soc 2008 14 10 1845 1852 10.1111/j.1532-5415.2008.01947.x 18811611 PMC2628807

[B42] Mezuk B Lohman M Dumenci L Lapane KL Are depression and frailty overlapping syndromes in mid- and late-life? A latent variables analysis Am J Geriatr Psych 2013 14 560 569 10.1016/j.jagp.2012.12.019 PMC3424389 23567406

[B43] Bergman H Ferrucci L Guralnik J Hogan DB Hummel S Karunananthan S Wolfson C Frailty: an emerging research and clinical paradigm–issues and controversies J Gerontol A BiolSci Med Sci 2007 14 7 731 737 10.1093/gerona/62.7.731 PMC2645660 17634320

